# Case Report: Genetic diagnosis and counseling for recurrent holoprosencephaly: a case of parental balanced translocation

**DOI:** 10.3389/fgene.2026.1761076

**Published:** 2026-10-21

**Authors:** Tingting Ge, Chuan Zhang, Yu Hao, Shibin Cheng, Bingbo Zhou, Xiaozhuan Wang, Yongwen Cao, Xiaojuan Lin

**Affiliations:** 1 Prenatal Diagnosis Center, Gansu Provincial Maternity and Child-care Hospital (Gansu Provincial Central Hospital), Key Laboratory of Maternal-Fetal Medicine and Reproductive Protection of Gansu Province, Lanzhou, China; 2 Center for Medical Genetics, Gansu Provincial Maternity and Child-care Hospital (Gansu Provincial Central Hospital), Gansu Provincial Clinical Research Center for Birth Defects and Rare Diseases, Lanzhou, China; 3 Planning and Finance Department, Gansu Provincial Maternity and Child-care Hospital (Gansu Provincial Central Hospital), Lanzhou, China

**Keywords:** case report, chromosomal translocation, holoprosencephaly, miscarriage, prenatal diagnosis

## Abstract

**Introduction:**

Holoprosencephaly (HPE) is a severe developmental anomaly of the brain, resulting from the failure of forebrain vesicle division during fetal development. Chromosomal abnormalities, such as trisomy 13 and trisomy 18, along with copy number variations (CNVs), are implicated in the pathogenesis of HPE. Furthermore, over 20 genes have been implicated in the pathogenesis of HPE, with SHH (MIM: 600725), ZIC2 (MIM: 603073), TGIF (MIM: 602630), and SIX3 (MIM: 603714) identified as the primary causative genes. This study aims to investigate the genetic causes underlying recurrent holoprosencephaly in a specific couple and to offer genetic counseling and reproductive guidance.

**Case Presentation:**

A 31-year-old woman with a history of 9 naturally conceived pregnancies, all ending in first-trimester miscarriage or fetal structural abnormalities. She is a balanced translocation, characterized by a chromosomal karyotype of 46,XX,t(3; 13)(q27; q31). In three instances, pregnancies advanced beyond 12 weeks and were complicated by fetal nuchal cystic hygroma and holoprosencephaly. In this study, prenatal diagnostic procedures were conducted on the last two pregnancies, identifying a duplication of approximately 12.74 Mb in the q27.2q29 region of chromosome 3 and a heterozygous deletion of approximately 21.78 Mb in the q31.3q34 region of chromosome 13 in the fetuses.

**Conclusion:**

In cases of recurrent miscarriages and abnormal fetal structures, it is imperative to perform karyotype analysis and related genetic tests. Upon elucidating the etiology, genetic counseling and reproductive interventions should be offered to mitigate the risk of chromosomally abnormal fetuses during pregnancy and to safeguard the physical and mental wellbeing of pregnant women.

## 1 Introduction

Holoprosencephaly (HPE) is a severe developmental anomaly of the brain, resulting from the failure of forebrain vesicle division during fetal development. It is characterized by the incomplete midline separation between the telencephalon and diencephalon (Ionescu et al., 2019). Recent advances in genetic research have elucidated that HPE is fundamentally a disorder arising from the dysregulation of multiple signaling pathways during early embryogenesis. This dysregulation predominantly affects the differentiation and patterning induction of the mesenchyme in the anterior forebrain region towards the ventral aspect of the cephalic neural tube (Golden, 1999). Given the shared genetic regulatory networks between facial structure and forebrain development, genetic factors are currently regarded as the primary etiological contributors to HPE, despite the presence of clear teratogenic exposures (e.g., maternal diabetes, alcohol) in some instances. Additionally, chromosomal abnormalities, such as trisomy 13 and trisomy 18, along with copy number variations (CNVs), are implicated in the pathogenesis of HPE (Malta, AlMutiri, Martin and Srour, 2023; Roessler and Muenke, 2010). Furthermore, over 20 genes have been implicated in the pathogenesis of HPE, with SHH (MIM: 600725), ZIC2 (MIM: 603073), TGIF (MIM: 602630), and SIX3 (MIM: 603714) identified as the primary causative genes (Bertolacini, Richieri-Costa and Ribeiro-Bicudo, 2010; Houtmeyers et al., 2016; Keaton et al., 2010; Solomon et al., 2009).

The ultrasonic diagnosis of holoprosencephaly during the first trimester is relatively straightforward, characterized by an indistinct anterior third of the brain midline on a transverse section of the fetal cranium, absence of the choroid plexus butterfly sign, presence of a single ventricle, and fused thalami. Notably, trisomy 13 is the most frequently associated chromosomal abnormality with holoprosencephaly (Wenghoefer et al., 2010).

Current guidelines for the genetic evaluation of recurrent pregnancy loss (RPL) and severe fetal structural anomalies recommend cytogenetic analysis of the products of conception (POC) and parental peripheral blood karyotyping to identify balanced structural rearrangements (Bender Atik et al., 2023; Practice Committee of the American Society for Reproductive Medicine, 2012). For patients with a history of recurrent pregnancy loss who experience another early pregnancy loss, they should be informed about the option of cytogenetic analysis of the products of conception to clarify the etiology and guide further management. Given that more than half of early pregnancy losses are caused by aneuploidy, in cases of recurrent pregnancy loss, *in vitro* fertilization with preimplantation genetic testing for aneuploidies can be considered to select embryos and transfer them into patients with proven fertility, thereby improving the efficiency of achieving an ongoing pregnancy (Sierra et al., 2025).

Carriers of balanced translocations typically present with normal phenotypes due to the absence of significant genetic material loss. However, they face a significantly elevated risk of spontaneous abortion, with reported rates as high as 57.8% for reciprocal translocation carriers and 43.6% for Robertsonian translocation carriers (Franssen et al., 2005). Given this high risk, current clinical guidelines, including those from the ESHRE and ASRM, recommend comprehensive genetic evaluation for couples with a history of two or more miscarriages or a pregnancy affected by severe fetal malformations (Bender Atik et al., 2023; Viotti et al., 2023).

This evaluation typically includes parental karyotyping to identify balanced structural rearrangements. Critically, for carriers identified through this process, preimplantation genetic testing for structural rearrangements (PGT-SR) is recommended as a key reproductive option. PGT-SR, performed on embryos created via *in vitro* fertilization (IVF), is specifically designed to distinguish between embryos with a normal karyotype, those carrying the balanced translocation (like the parent), and those with unbalanced chromosomal content that would likely lead to miscarriage or an affected pregnancy (Bender Atik et al., 2023). It is important to differentiate PGT-SR from preimplantation genetic testing for aneuploidy (PGT-A). While PGT-A screens embryos for whole-chromosome gains or losses, it is not optimized to detect the segmental imbalances resulting from a parental translocation. Therefore, PGT-SR is the appropriate and recommended test for translocation carriers seeking to reduce their risk of recurrent.

This study conducted a retrospective analysis of a case involving multiple pregnancies complicated by severe fetal brain malformations (holoprosencephaly) in a carrier of a maternal balanced translocation, aiming to elucidate the pathogenic cause, discuss the genotype-phenotype correlation, and underscore the importance of timely genetic evaluation and counseling.

## 2 Case report

A 31-year-old woman with a history of 9 naturally conceived pregnancies, all ending in first-trimester miscarriage or fetal structural abnormalities. Both she and her partner were healthy, unrelated, and had no exposure to toxins or radiation. The family had a history of recurrent miscarriages (Figure 1). In her fifth pregnancy in 2021, a 12-week Nuchal Translucency (NT) scan indicated fetal holoprosencephaly, cystic hygroma, and generalized skin edema (Figure 2), leading to pregnancy termination without genetic testing. Subsequent pregnancies also ended in miscarriage. In her eighth pregnancy in 2023, a 12-week NT scan showed fetal cystic hygroma and abnormal cardiac structure, which was later confirmed as cystic hygroma, pleural effusion, and a single ventricle at 15 weeks (Figure 3). CNV-Seq analysis identified a 12.62 Mb duplication in the q27.2-q29 region of fetal chromosome 3 (chr3:g.185220000_197840000) and a 21.78 Mb deletion in the q31.3-q34 region of chromosome 13 (chr13:g.93320000_115100000) (Figure 4). Karyotype analysis indicated a chromosomal composition of 46,XY,del(13)(q31) (Figure 5). The maternal karyotype was determined to be 46,XX,t(3; 13)(q27; q31) (Figure 6), while the paternal karyotype was normal. In her ninth pregnancy in 2024, a 12-week NT scan revealed fetal cystic hygroma, generalized skin edema, and holoprosencephaly (Figure 7). Following the induction of labor, trophoblast whole-exome sequencing (tro-WES) was performed at an external medical facility. The results identified a 12.74 Mb duplication at chromosome 3q27.2-q29 and a heterozygous deletion of approximately 21.78 Mb at chromosome 13q31.3-q34 (Figure 8; Table 1). These findings were consistent with the fetal testing results obtained in 2023. This study received approval from the hospital’s Ethics Committee (No.2024GSFY07).

**FIGURE 1:**
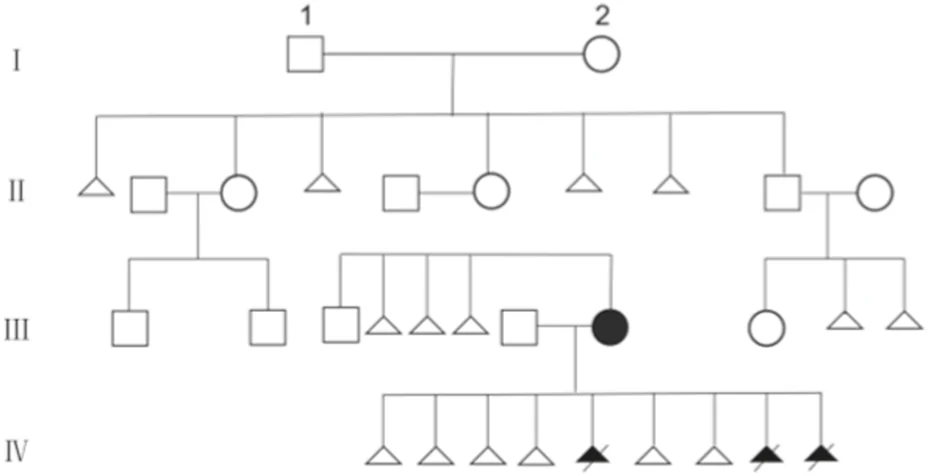
Patient’s family chart.

**FIGURE 2:**
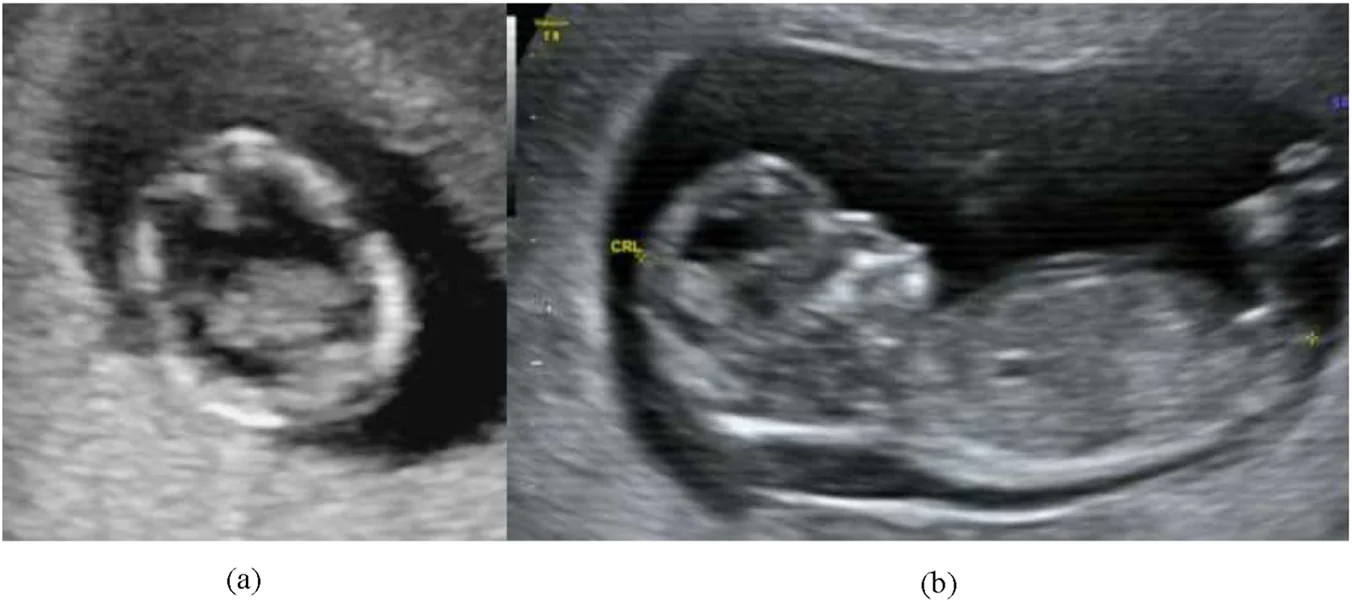
Fifth pregnancy, 12-week NT scan. **(a)** Holoprosencephaly, **(b)** Cystic hygroma.

**FIGURE 3:**
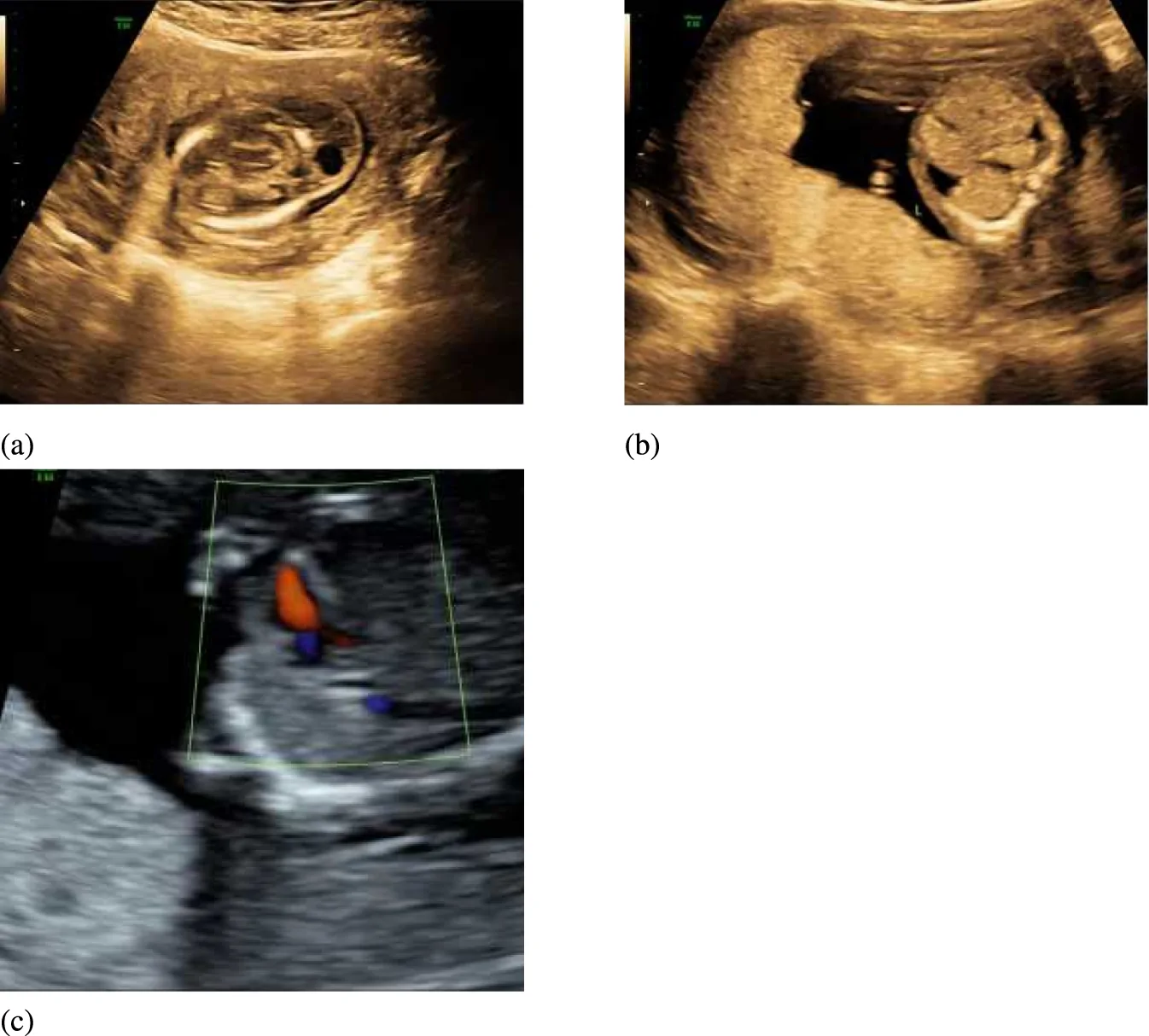
Eighth Pregnancy, 15-week ultrasound scan. **(a)** Cystic hygroma, **(b)** Pleural effusion, **(c)** Single ventricle.

**FIGURE 4:**
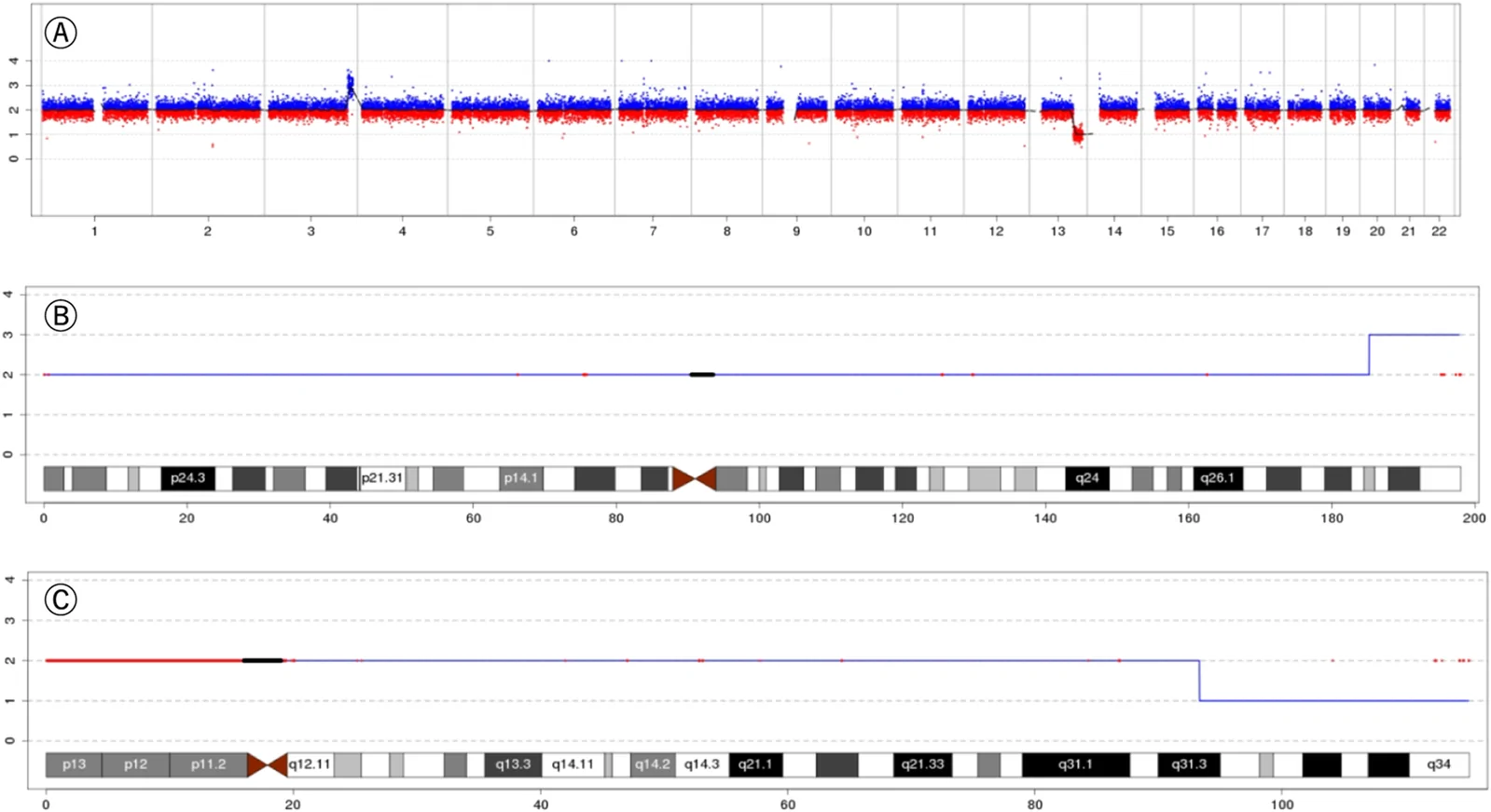
CNV-Seq test result of the eighth pregnancy: **(A)** Whole genome results; **(B)** Chromosome 3 test results; **(C)** Chromosome 13 test results.

**FIGURE 5:**
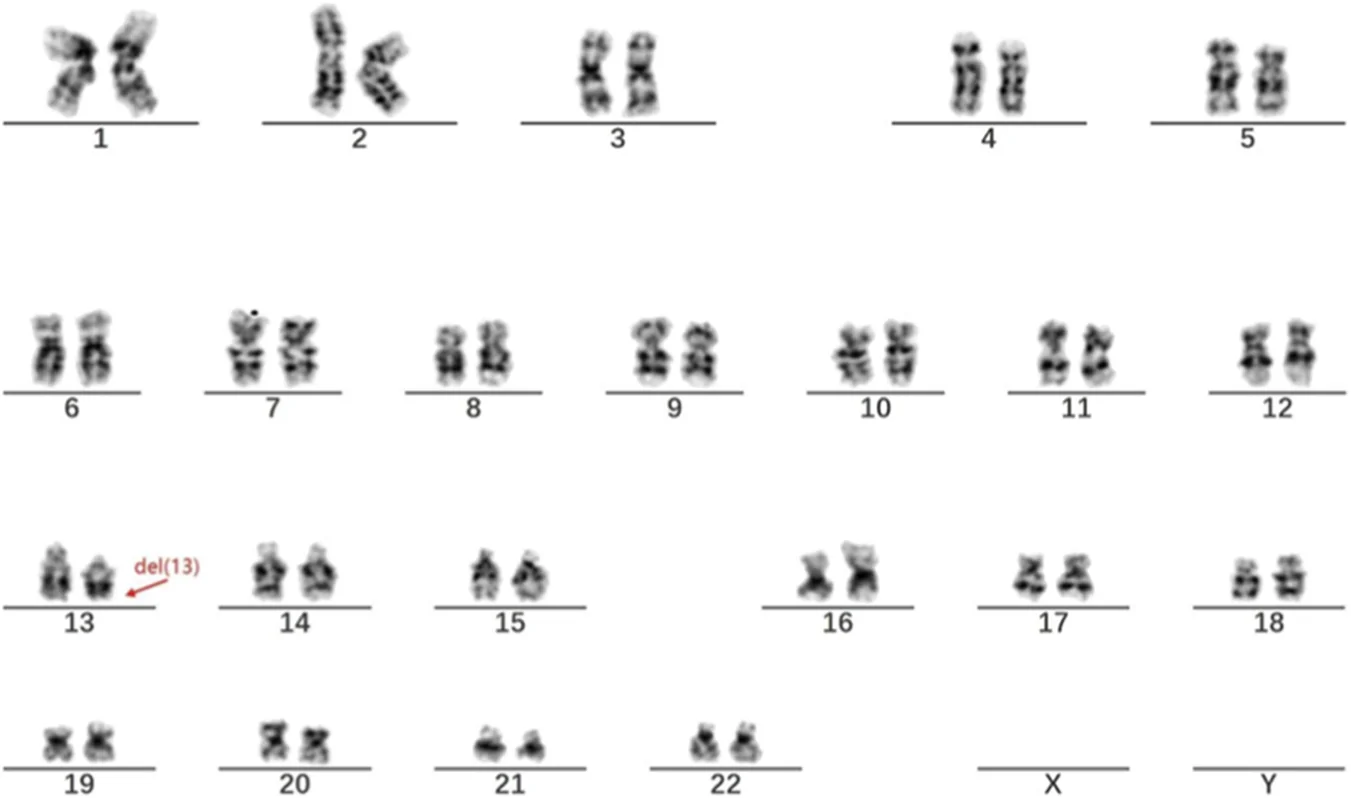
Fetal amniotic fluid karyotype analysis results of the eighth pregnancy.

**FIGURE 6:**
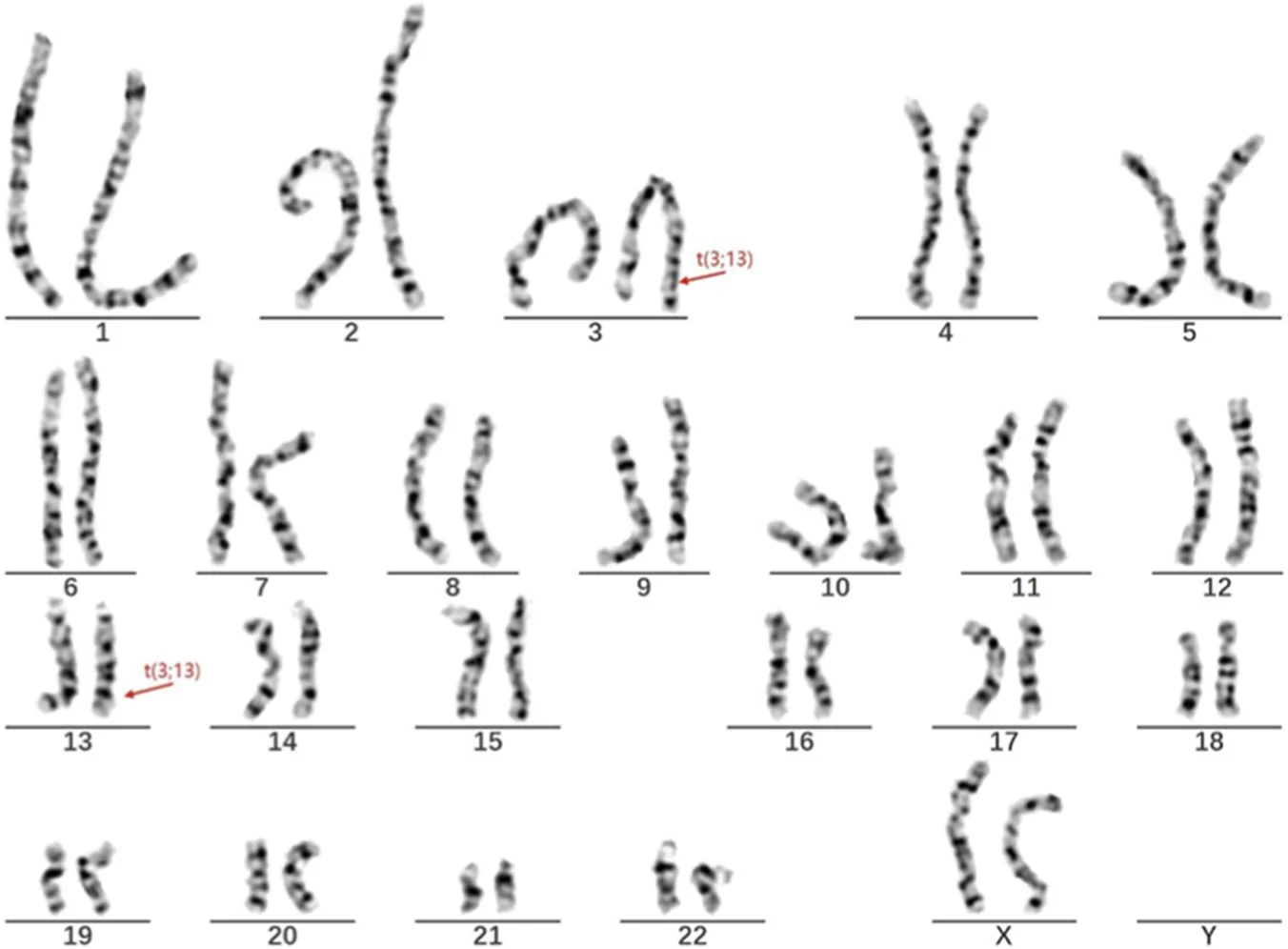
High-resolution chromosomal karyotyping results from maternal peripheral blood.

**FIGURE 7:**
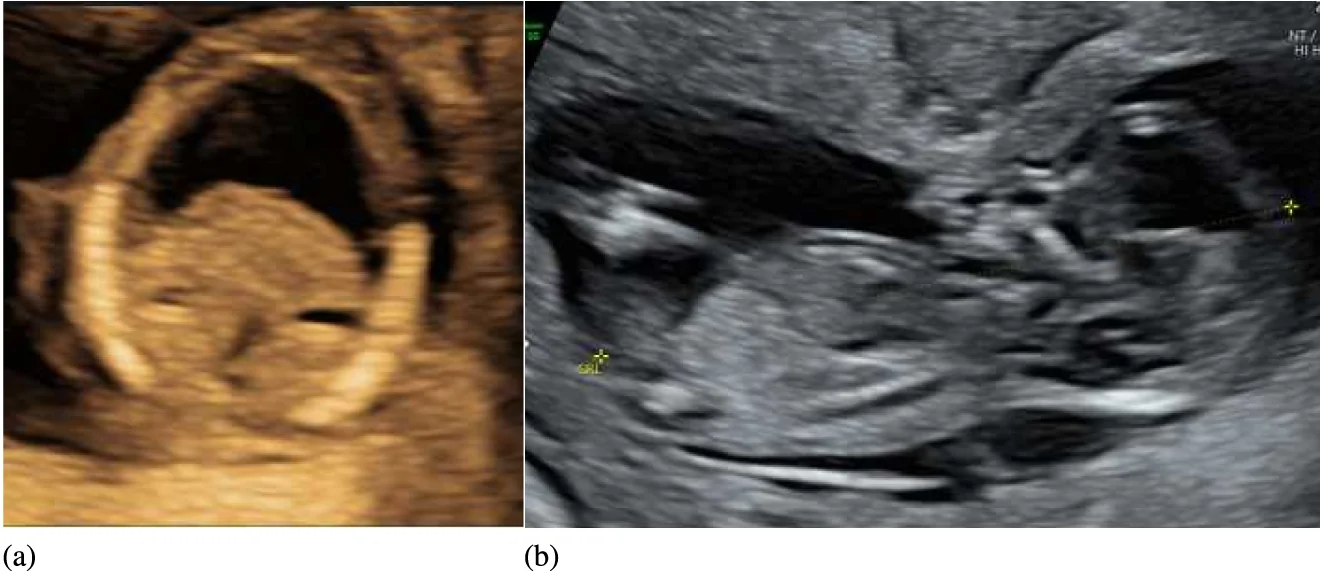
Ninth pregnancy, 12-week NT scan. **(a)** Holoprosencephaly, **(b)** Cystic hygroma with systemic edema.

**FIGURE 8:**
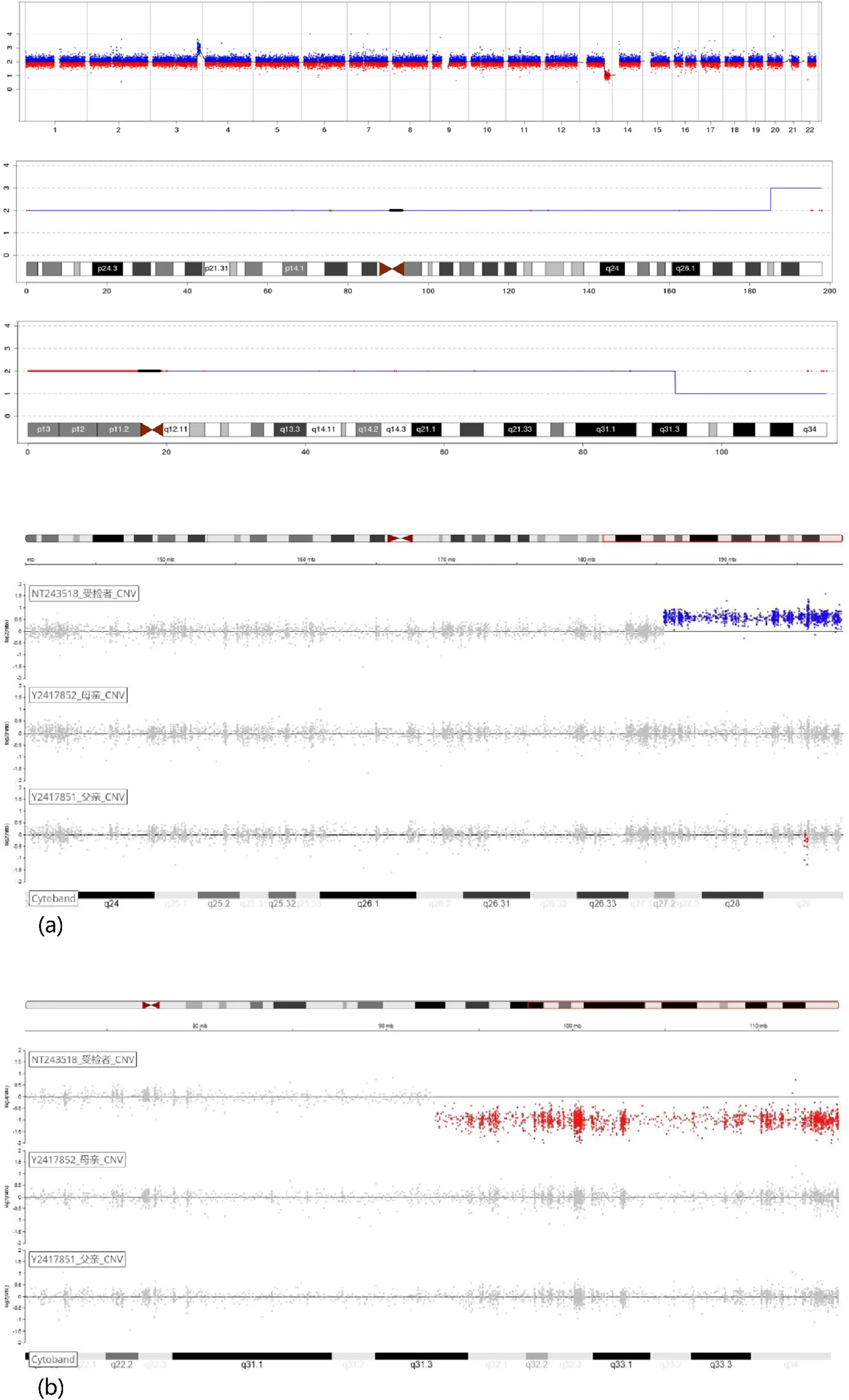
Ninth pregnancy, tro-wes analysis results (fetus and parents). **(a)** IGV schematic diagram of the 3q27.2q29 Region for the Subject and Parents; **(b)** IGV schematic diagram of the 13q31.3q34 region for the subject and parents.

**TABLE 1:** Summary of pregnancy characteristics, prenatal findings, genetic testing and outcomes.

Pregnancy no.	Gestational age (week)	Ultrasound findings	Genetic testing	Outcome
1–4	<10	Embryonic demise	NO	SAB
5	12	Holoprosencephaly, Cystic hygroma, Generalized skin edema	NO	TOP
6–7	<10	Embryonic demise	NO	SAB
8	12	Cystic hygroma, Cardiac abnormality	Chr3:g.185220000-197840000dup (2.62 Mb) Chr13:g.93320000-115100000del (21.78 Mb)	TOP
	15	Cystic hygroma, Pleural effusion, Single ventricle		
9	12	Cystic hygroma, Generalized skin edema, Holoprosencephaly	Chr3:g.185220000-197840000dup (12.74 Mb) Chr13:g.93320000-115100000del (21.78 Mb)	TOP

SAB: spontaneous abortion; TOP: termination of pregnancy.

## 3 Discussion

The subject of this study is a female carrier of a balanced translocation, characterized by a chromosomal karyotype of 46,XX,t(3; 13)(q27; q31). This individual has experienced multiple pregnancies, the majority of which have culminated in embryonic arrest during the first trimester. In three instances, pregnancies advanced beyond 12 weeks and were complicated by fetal nuchal cystic hygroma and holoprosencephaly. In this study, prenatal diagnostic procedures were conducted on the last two pregnancies, identifying a duplication of approximately 12.74 Mb in the q27.2q29 region of chromosome 3 and a heterozygous deletion of approximately 21.78 Mb in the q31.3q34 region of chromosome 13 in the fetuses.

The deletion in chr13q31.3-q34 encompasses 71 protein-coding genes, including the entire ZIC2 gene (OMIM: 603073)—a well-established causative gene for HPE (Barratt et al., 2018; Keaton et al., 2010). ZIC2 encodes a zinc finger transcription factor essential for early forebrain development, particularly in cell signaling and pattern formation during embryogenesis (Barratt et al., 2018). Haploinsufficiency of ZIC2 (resulting from the chr13q deletion in this case) is known to cause HPE (OMIM: 609637), along with associated phenotypes such as intellectual disability, craniofacial dysmorphisms, and neural tube defects (Barratt et al., 2018; Pineda-Alvarez et al., 2011). This genotype-phenotype correlation explains the recurrent fetal HPE observed in this case.

Conversely, the 12.62 Mb duplication at chromosome 3q27.2-q29 encompassing 69 protein-coding genes. While this region encompasses the locus for the 3q29 microduplication syndrome (typically ∼1.6 Mb), the present duplication is considerably larger. Phenotypes associated with large 3q duplications are variable and can include intellectual disability, microcephaly, and congenital heart defects (Fernández-Jaén et al., 2014). The phenotypic expression of this syndrome exhibits variability among patients (Kashevarova et al., 2025). In our study, fetal cardiac structural abnormalities were observed in one instance. However, given the early gestational age at which the ultrasound was conducted, the diagnosis may not be conclusive due to the limited gestational development at the time of examination.

In our case, the pregnant woman experienced multiple miscarriages without the benefit of genetic testing on the aborted tissues. Following her eighth pregnancy, she underwent interventional prenatal diagnosis, which revealed through CNVs a duplication in the region of chromosome 3q27.2-q29 and a deletion in chromosome 13q31.3-q34. Karyotype analysis indicated a karyotype of 46, XY, del(13)(q31). The 3q duplication was not visible by conventional G-banding due to its subtelomeric location and the resolution limits of karyotyping, which typically detects imbalances >5–10 Mb. It was hypothesized that one of the fetus’s parents might possess a chromosomal translocation. High-resolution banding karyotype analysis subsequently identified the pregnant woman’s chromosomal karyotype as 46, XX, t(3; 13)(q27; q31), while her husband’s karyotype was normal.

This study reports a case of recurrent miscarriage associated with a maternal balanced translocation. Couples with recurrent pregnancy loss should be offered cytogenetic analysis. When a chromosomal abnormality is identified, genetic counseling is essential (Bender Atik et al., 2023; Practice Committee of the American Society for Reproductive Medicine, 2012). In this case, the absence of timely genetic counseling led to nine consecutive unsuccessful pregnancies, resulting in significant physical and psychological burden. Had fetal genetic testing been performed after the second miscarriage to identify the cause, followed by the use of preimplantation genetic testing to select embryos, it might have reduced the occurrence of subsequent miscarriages and stillbirths, thereby potentially altering the clinical outcome.

Therefore, after two or more consecutive embryonic arrests, timely genetic testing of abortus tissues is recommended to determine the etiology and prevent similar outcomes. For couples identified as carriers of balanced translocations, genetic counseling should include the option of preimplantation genetic screening to reduce the risk of miscarriage and stillbirth.

## Data Availability

The datasets presented in this study can be found in online repositories. The names of the repository/repositories and accession number(s) can be found in the article/supplementary material.
